# Household costs for personal protection against mosquitoes: secondary outcomes from a randomised controlled trial of dengue prevention in Guerrero state, Mexico

**DOI:** 10.1186/s12889-017-4303-y

**Published:** 2017-05-30

**Authors:** José Legorreta-Soberanis, Sergio Paredes-Solís, Arcadio Morales-Pérez, Elizabeth Nava-Aguilera, Felipe René Serrano-de los Santos, Belén Madeline Sánchez-Gervacio, Robert J. Ledogar, Anne Cockcroft, Neil Andersson

**Affiliations:** 1 Centro de Investigación de Enfermedades Tropicales de la Universidad Autónoma de Guerrero, Guerrero state, Acapulco, Mexico; 2 CIETinternational, New York, NY USA; 3 Department of Family Medicine, McGill University, Montreal, Canada; 4 CIET Trust, Gaborone, Botswana

**Keywords:** Dengue prevention, Personal protection costs, Insecticide anti-mosquito products

## Abstract

**Background:**

Dengue is a serious public health issue that affects households in endemic areas in terms of health and also economically, imposing costs for prevention and treatment of cases. The Camino Verde cluster-randomised controlled trial in Mexico and Nicaragua assessed the impact of evidence-based community engagement in dengue prevention. The Mexican arm of the trial was conducted in 90 randomly selected communities in three coastal regions of Guerrero State. This study reports an analysis of a secondary outcome of the trial: household use of and expenditure on anti-mosquito products. We examined whether the education and mobilisation activities of the trial motivated people to spend less on anti-mosquito products.

**Methods:**

We carried out a household questionnaire survey in the trial communities in 2010 (12,312 households) and 2012 (5349 households in intervention clusters, 5142 households in control clusters), including questions about socio-economic status, self-reported dengue illness, and purchase of and expenditure on insecticide anti-mosquito products in the previous month. We examined expenditures on anti-mosquito products at baseline in relation to social vulnerability and we compared use of and expenditures on these products between intervention and control clusters in 2012.

**Results:**

In 2010, 44.2% of 12,312 households reported using anti-mosquito products, with a mean expenditure of USD4.61 per month among those who used them. Socially vulnerable households spent less on the products. In 2012, after the intervention, the proportion of households who purchased anti-mosquito products in the last month was significantly lower in intervention clusters (47.8%; 2503/5293) than in control clusters (53.3%; 2707/5079) (difference − 0.05, 95% CIca −0.100 to −0.010). The mean expenditure on the products, among those households who bought them, was USD6.43; 30.4% in the intervention clusters and 36.7% in the control clusters spent more than this (difference − 0.06, 95% CIca −0.12 to −0.01). These expenditures on anti-mosquito products represent 3.3% and 3.8% respectively of monthly household income for the poorest 10% of the population in 2012.

**Conclusions:**

The Camino Verde community mobilisation intervention, as well as being effective in reducing dengue infections, was effective in reducing household use of and expenditure on insecticide anti-mosquito products.

**Trial registration:**

(ISRCTN27581154).

## Background

Since its re-emergence in the Americas, dengue has continued to spread in all its clinical forms, despite vector-control efforts on the part of health services from every country in the region, and health services in Latin America use considerable resources to treat cases of dengue fever [1].

In Mexico, the Specific Action Program for Dengue 2013–2018 (*Programa de Acción Específico. Prevención y Control de Dengue 2013–2018*) confirmed a protocol to identify dengue fever patients as potential carriers of the disease, and to register them in the National Epidemiological Surveillance System (*Sistema Nacional de Vigilancia Epidemiológica*) to locate cases in time and space. The programme includes guidelines for prevention and control measures to be carried out during visits by health workers to patients’ homes and neighboring households, during which a larvicide, temephos, is placed in water containers, and the area surrounding each home is fumigated [2].

Studies in Latin America have estimated the direct and indirect costs of in- and out-patient dengue cases, workdays lost due to the disease, and disability- or quality-adjusted life years [3–7]. Authors have reported on the household costs of actions to prevent mosquito-borne infections in Asia and Africa [8–12]. However, we have not found any published report of a randomised controlled trial of dengue prevention that examined the impact of the trial intervention on household expenditures on prevention. The study reported here describes household expenditures on personal protection measures for dengue prevention and examines the impact on these expenditures as a secondary outcome of the Mexican arm of the Camino Verde trial of community-based activities for dengue prevention, undertaken in Mexico and Nicaragua [13]. Our analysis examines whether Camino Verde’s education and mobilisation activities helped to motivate people to control mosquito breeding in their homes and neighbourhoods by non-chemical means and to spend less on personal protection measures.

## Methods

The methods of the Camino Verde trial are described in detail elsewhere [13, 14]. This study is based on findings from the Mexican arm of the trial. The study included a random sample of 90 communities from the most recent census in the three coastal regions in the state of Guerrero, Mexico. After the 2010 baseline household survey, we stratified clusters according to evidence of recent dengue virus infection in children aged 3–9 years and vector indices, and allocated half to receive the intervention. The intervention encouraged community mobilisation using results from the baseline survey. Each intervention cluster adapted the basic intervention - chemical-free prevention of mosquito reproduction - to its own circumstances. However, the government-run dengue control programme, including temephos application to household water containers and fumigation, continued in all clusters. The impact survey of the trial took place in 2012. Both the baseline and the impact survey included a household questionnaire, an entomological survey of larvae and pupae of *Aedes aegypti* in water containers, and paired saliva samples in children aged 3–9 years to look for evidence of recent dengue infection (doubling of specific IgG levels). The primary trial outcomes included self-reported dengue cases in the past year, serological evidence of recent dengue virus infection, and conventional entomological indices of *Aedes aegypti* infestation. A secondary outcome measure in the trial was household expenditure on personal protection measures for dengue prevention.

### Estimation of personal protection expenditures

The household questionnaire in the baseline and impact surveys included questions about use of anti-mosquito products such as insecticide sprays or spirals, the frequency of their use, and the amount spent on these products during the month before the survey.

We estimated the average monthly expenditure per household on insecticides, among households that reported any expenditure on these products during the last month. We also estimated the mean expenditure in the last month across all households, including those who spent nothing. In the surveys the amounts spent were reported in Mexican pesos (MXN). During the years 2010 to 2012 the US dollar (USD) exchange rate for the Mexican dollar fluctuated around MXN13.00 to USD1.00 so we have used that rate for convenience here [15].

Using the data from the baseline survey, we compared the average monthly expenditure on anti-mosquito insecticides between groups of households according to characteristics potentially related to the use of these products: being covered by the healthcare services’ temephos (Abate®) distribution programme; reporting at least one case of dengue illness in the previous year; and having evidence of recent dengue infection among children aged 3–9 years. We also examined expenditure on insecticides according to six household social vulnerability characteristics: socioeconomic region; area of residence; ethnicity; household type; education level of the household head; and employment status of the household head. We considered households to be more vulnerable if they were: located in the Costa Chica region; located in rural areas; inhabited by indigenous people; buildings with impermanent construction; headed by someone with a third-grade education or lower; or headed by someone unemployed.

### Extrapolation of expenditure figures

We extrapolated from expenditures reported by households in the sample in the baseline survey to estimate expenditure on insecticide anti-mosquito products by the whole population of Guerrero State’s three coastal regions. We estimated the number of inhabited households per region by dividing the population of the region, from the census by the state average of 4.2 people per household [16]. We applied the proportion of households who reported spending on insecticides in the baseline sample to the estimated number of households in the regions, then calculated the mean total expenditure per region by multiplying by the reported monthly expenditure in the sample households who reported expenditure. To estimate annual expenditure in each region we multiplied the monthly figure by 12. The baseline survey was carried out between January and June 2010, and the reported monthly expenditure in these months would be expected to be relatively low as it is not the main season for mosquitoes. For a more conservative estimate, we multiplied the monthly expenditure by 6, on the assumption that there may be little or no expenditure for six months of the year.

### Analysis

Trained operators entered data, using EpiData software, with double data entry and validation to minimise keystroke errors. Analysis relied on the public domain software CIETmap [17, 18]. We calculated the mean and standard deviation (SD) for reported monthly household expenditure on insecticides and tested the significance of differences in expenditures between sub-groups using an unpaired t-test or the Kruskal-Wallis non-parametric test for sample difference when variances were different between the groups. We tested the significance of the associations between household reported insecticide use (yes or no) and self-reported dengue cases and serologically-defined dengue infection, using the Mantel-Haenszel procedure and reporting the Odds Ratio (OR) and cluster-adjusted 95% confidence intervals (95% CIca) [19, 20].

From the impact survey, we tested the significance of differences between intervention and control clusters in proportions of households reporting expenditure on insecticides, and in proportions of spending households, and of all households, spending more than the mean amount on these products in the last month, using a cluster t-test. We treated each cluster as a unit in an intention-to-treat analysis (everyone in each cluster, all clusters per allocation) [21].

## Results

In the baseline survey we analysed data from 12,312 households. We excluded 87 of the 12,399 responses (0.7%), because they came from exclusively commercial establishments (stores, workshops, etc.). The impact survey included 10,491 households, 5349 in 45 intervention sites and 5142 in 45 control sites. The reduced number of households in the impact survey was related to the deteriorating security situation in Guerrero State; some people had moved out of the country or to other, more secure, communities. The interviewers encountered more empty houses in the follow up survey than in the baseline.

### Baseline survey

#### Personal protection costs at baseline

In the baseline survey 44.2% (5433/12,287) of households reported using insecticide anti-mosquito products such as sprays and spirals. The average monthly expenditure for these products was USD 4.86 (SD 4.66). A quarter (24%; 1293/5407) of households reported using anti-mosquito products daily, 20% (1074/5407) used them two or three times a week, 19% (1019/5407) used them once a week, and 37% (2021/5407) used them every two or more weeks. As expected, the reported monthly expenditure varied by reported frequency of use. It was USD6.53 (*n* = 1281; SD 7.97) for daily use, USD5.35 (*n* = 1061; SD 4.41) for use two or three times per week, USD4.45 (*n* = 1007; SD 3.83) for weekly use, and USD3.73 (*n* = 1990; SD 3.66) for use every two or more weeks. The difference in expenditures between groups with different frequency of use was statistically significant (Kruskal-Wallis H 394; df 3; *p* < 0.000001).

The extrapolation of expenditures from the sample to the whole population of the coastal regions in Guerrero state is shown in Table 1. Expenditure was highest in Acapulco region because of the higher number of households and also the higher proportion of households reporting insecticide use.

**Table 1:** Estimated expenditure in USD on insecticides in the three coastal regions of Guerrero State in 2010

	Acapulco	*C. Grande*	*C. Chica*
Estimated number of inhabited households (population/4.2)	188,088	98,522	102,024
Estimated number of households using insecticides^a^	96,865	39,901	40,801
Amount spent on insecticides in the last month, among those using them^b^	491,776	168,811	213,420
Amount spent on insecticides in the last year^c^	5,901,312	2,205,732	2,561,040

^a^The proportions of households that reported using insecticides in the 2010 baseline study were: Acapulco region 51.5%, Costa Grande region 40.5% and Costa Chica region 40%

^b^The mean expenditures on insecticides in the last month were: Acapulco USD5.0; Costa Grande USD4.2; and Costa Chica USD5.2.

^c^The amount shown is calculated by multiplying the monthly amount by 12. A more conservative estimate, assuming the products are not purchased for roughly half the year, multiplies the monthly amount by 6, and gives the following annual expenditures: Acapulco USD2,950,656; C. Grande USD1,012,866; C. Chica USD1,280,520

In the baseline survey, 6.3% of households (780/12,308) reported at least one case of dengue illness in the last 12 months. The rate of self-reported dengue cases was 8.3% (452/5433) among households reporting insecticide use, and 4.8% (327/6850) among households not reporting insecticide use. Households that reported insecticide use were 80% more likely to report a case of dengue illness in the last year than households that did not report insecticide use (OR 1.81; 95% CIca 1.53–2.10). Among households who used insecticide products, the average monthly expenditure was significantly lower among households that did not report any dengue cases in the last year (USD4.80; *n* = 4905; SD 4.53), than among households that reported one or more cases of dengue illness (USD5.53; *n* = 448; SD 5.9) (Kruskal-Wallis H 4.4; df 1; *p* = 0.03).

Some 10.5% (1284/12,251) of households reported that government workers had never placed temephos in their water containers; 33% (4063/12,251) of households reported receiving temephos in the last month; 53% (6566/12,251) received temephos two months ago or more; and 3% (338/12,251) could not specify when they received temephos. In households which had never been covered by the temephos programme, 36% (461/1284) reported using insecticide anti-mosquito products, while among those that had received temephos at least once the rate was 45% (4955/10,967). Households which were not covered by the temephos programme were 32% less likely to have used insecticide products compared with those which were covered by the programme (OR =0.68; 95%CIca 0.55–0.84). Among households using insecticide products, the average monthly expenditure was not significantly different between those with temephos coverage (4.9 USD; *n* = 4888; SD 4.6) and those without it (4.9 USD; *n* = 449; SD 5.1; Kruskal-Wallis H 0.968; df1; *p* = 0.33).

#### Expenditures and social vulnerability

There were significant differences in the average monthly expenditure on insecticides based on household social vulnerability characteristics (Table 2). Expenditures were higher in Acapulco region than in the other two regions. Expenditures were generally lower among more vulnerable households. Rural households and those with a non-permanent construction spent less than urban households or those with a permanent construction. Households with a less educated head or an unemployed head spent less than those with a more educated head or an employed head.

**Table 2:** Average monthly household expenditure on insecticides in USD by social vulnerability characteristics in 2010 baseline survey

Characteristic	n=	% of households using insecticides	Mean expenditure last month	SD	p=
Acapulco	2189	51.5	5.1	4.8	<0.0000001
Costa Grande	1618	40.5	4.2	3.9	
Costa Chica	1551	40.0	5.2	5.2	
Rural	2655	40.4	4.6	4.4	<0.0000001
Urban	2703	48.7	5.1	4.9	
Indigenous	253	56.5	5.1	4.4	0.21
*Mestizos*	5092	43.7	4.9	4.7	
Non-permanent house	626	36.5	4.5	4.1	0.00002
Semi-permanent house	2104	41.6	4.7	4.8	
Permanent house	2610	49.2	5.0	4.7	
Household head education:					
Less than 3rd grade	1651	38.1	4.5	4.5	<0.0000001
4th grade to high-school	3156	46.4	4.9	4.7	
Technical school or higher	499	56.1	5.6	5.2	
Household head unemployed	776	40.3	4.6	4.9	0.00009
Household head employed	4570	45.0	4.9	4.6	

### Impact of the trial intervention

Table 3 shows the proportions of households reporting purchase of anti-mosquito products in the last one month and the mean expenditure on these products: among the households who bought them, and among all households. In both the intervention and control groups the proportion of households buying anti-mosquito products and the amount they spent on them are higher than in the baseline survey (see Table 2), reflecting the timing of the surveys: the 2010 baseline took place in the “low season” for mosquitos (January to May 2010), and the 2012 impact survey took place in the “high season” for mosquitos in August–November.

**Table 3:** Proportion of households that purchased anti-mosquito products, and expenditure during the last month among those who purchased the products, in trial intervention and control sites surveyed in August–November 2012

	Intervention clusters	Control clusters	Difference of proportions (95%CIca)
Surveyed households	5349	5142	
Proportion of households that purchased anti-mosquito products^a^	47.8% (2530/5293)	53.3% (2707/5079)	−0.05 (−0.1 to −0.01)
*Among households spending anything*			
Mean expenditure in the last month (USD)	6.0 (SD 5.9)	6.83 (SD 6.84)	
Proportion spending more than the mean of USD 6.43^b^	30.4% (768/2530)	36.7% (993/2707)	−0.06 (−0.12 to −0.01)
*Among* *all* *households*			
Mean expenditure in the last month (USD)	2.86 (SD 5.12)	3.65 (SD 6.04)	
Proportion spending more than the mean of USD 3.25^c^	30.6% (1622/5293)	37.5% (1906/5079)	−0.07 (−0.09 to −0.05)

^a^Cluster t-test. *t* = −2.193, df 88 *p* = 0.031

^b^Cluster t-test. *t* = −1.978, df 88, *p* = 0.05

^c^Cluster t-test. *t* = −2.653, df 88, *p* = 0.009

Table 3 shows that after the intervention, the proportion of households that purchased anti-mosquito products in the last month was significantly lower in the intervention sites (48%) than in the control sites (53%). At the time of the 2010 baseline, 43.2% (2647/6130) of households in clusters that subsequently received the intervention used insecticide anti-mosquito products, compared with 45.2% in clusters that subsequently served as controls; the difference was not statistically significant (cluster t-test, *t* = 0.515, *p* = 0.608). In 2012, the mean monthly expenditure on these products, among households that purchased them, was lower in the intervention sites than in the control sites, and the proportion spending more than the mean was significantly lower in the intervention sites (Table 3). Also in 2012, the mean monthly expenditure on anti-mosquito products across all households (including those not spending anything) was lower in the intervention sites, and the proportion spending more than this mean amount was significantly lower in the intervention sites (Table 3).

## Discussion

Our study shows that expenses for the purchase of products for personal protection against mosquitoes are an important proportion of monthly household incomes in Guerrero state**.** According to the 2012 National Household Income and Expenditure Survey, the average monthly income for Mexican households in the lowest income decile was USD171 [22]. The monthly expenditures on insecticide anti-mosquito products reported in our 2012 impact survey, of USD6.0 in intervention communities and USD6.83 in reference communities, represent 3.3% and 3.8% respectively of monthly income for people in this decile.

The findings from our study add to the existing literature on household expenditures on anti-mosquito products, as a means of protection against dengue and other mosquito-borne diseases. Mulla and colleagues estimated an expenditure between USD13.75 and USD86.13 on anti-mosquito products per household per year in four communities in Thailand, and reported that these expenses represented between 0.3% and 0.7% of the annual household income in Thailand [8]. A 2003 study in The Gambia reported that most (81%) of the recurring household expenditure for malaria protection was on insecticide anti-mosquito products rather than on bed nets [23]. Another 2003 survey in the Pondicherry region of Southern India reported that 99% of urban dwellers and 73% of rural dwellers used insecticide anti-mosquito products at some time in the year, and that annual expenditure on these products in urban areas was 0.63% of annual per capita income [9]. Similarly, a survey in Jaffna district, Sri Lanka, reported that 96% of respondents spent funds on products for personal protection against mosquitoes, mainly spirals, with monthly expenditure between USD0.70 and USD12.53 [10]. A survey in Orissa, India, reported use of anti-mosquito products by 99% of urban and 84% of rural households, with an average monthly expenditure of USD8.13 in urban areas and USD5.90 in rural areas [11]. In north-eastern Tanzania, a survey reported that households spent an average of USD0.18 on bed nets and their treatment each fortnight (47% of total prevention costs) and USD0.21 on insecticide anti-mosquito products (50% of the total) [12]. A 2012 survey of household incomes and expenditures in Mexico reported a household quarterly expenditure of USD21.83 (USD7.28 per month) on insecticide anti-mosquito products [22].

Table 4 summarises the monthly expenditure on anti-mosquito products reported by other authors in other countries. Using a Purchasing Power Parity conversion factor [24], the monthly expenditure estimates in our study fit within the range of expenditures previously reported.

**Table 4:** Summary of monthly household expenditures on anti-mosquito products reported by other authors

Author, country and year	Monthly expenditure reported (USD)	Equivalent in 2012 USD	Purchasing Power Parity conversion factor	Purchasing Power Parity expenditure
Mulla et al. Thailand 1999 [8]	4.00–25.00	5.50–34.45	0.4	13.75–86.13
Wiseman et al. Gambia 2003 [23]	2.50	3.13	0.3	10.43
Surendran et al. Sri Lanka 2007 [10]	0.19–3.40	0.21–3.76	0.3	0.70–12.53
Babu et al. India 2007 [11]	1.60–2.20	1.77–2.44	0.3	5.90–8.13
McElroy et al. Tanzania 2009 [12]	0.42	0.44	0.4	1.10
ENIGH, Mexico 2012 [22]	7.28	7.28	0.6	12.13

In the baseline survey, we found an association between a self-reported case of dengue illness in the household and a greater likelihood of the household purchasing insecticide anti-mosquito products. We have to be cautious in interpreting this finding from a cross-sectional enquiry. It could be that the response of the health services to a case of dengue, which includes placing temephos into water containers in the index household and surrounding households, as well as fumigation of the area, encourages the residents to use more anti-mosquito products.

Our finding of more expenditure on insecticides with more education of the household head (see Table 2) runs contrary to the idea that more educated households would be more aware of the health dangers of insecticides so would use them less. However, there is little concern about toxicity of insecticide products in Mexico [25] and the higher expenditure on such products when the household head is educated probably reflects the better economic status of such households. Other authors have also reported more expenditure on insecticide products when the household head is more educated [12]. A small study in Sri Lanka found no association between level of education and awareness about mosquito-borne diseases [26]. As described in the main report of the Camino Verde trial, there was a small but significant increase in knowledge of the dengue vector related to the intervention; but the impact survey questionnaire did not ask about knowledge of health effects of insecticides [13].

A key aim of our study was to estimate the impact of the Camino Verde trial on a stated secondary outcome of the trial: use of and expenditure on insecticide anti-mosquito products. Our findings indicate that after the trial intervention, fewer households in the intervention clusters purchased insecticide anti-mosquito products, and those who did buy them spent less on them than did households in the control clusters. Discouraging the use of insecticide sprays or spirals as protection against mosquitoes and the illnesses they are associated with, such as dengue, was not an explicit activity in the intervention design. However, community educators, called *brigadistas*, encouraged reflection and dialogue about various options for controlling mosquitoes, and the emphasis in the educational messages, which involved showing householders where mosquito larvae and pupae were lurking on their own premises and explaining to them how the mosquito development cycle could be interrupted, was on non-chemical solutions. The cost implications of the brigadistas’ interventions and discussions with householders are considered in another article about the Camino Verde trial [27].

Our finding that vulnerable households spent less on insecticides for personal protection against mosquitoes (see Table 2) could suggest that these personal protection measures might be too expensive for the poorest section of the population. Non-chemical control methods are accessible to all households, and are an effective means of prevention [28].

## Conclusion

The evidence from this study supports the hypothesis that the participatory intervention of Camino Verde, based on bringing the community voice to action for dengue prevention, can lead to a more sustainable physical and biological control of the *Aedes aegypti* vector, with a lower number of houses purchasing anti-mosquito products and less expenditure on these products. Added benefits of reduced reliance on insecticides for mosquito control are a reduction in their potential to harm human health and the environment. The household resources saved can be available to meet other household needs.

## Abbreviations

95% CI: 95% confidence interval
95% CIca: 95% confidence interval cluster adjusted
MXN: Mexican Peso
USD: US dollar
